# An experimental study of respiratory aerosol transport in phantom lung bronchioles

**DOI:** 10.1063/5.0029899

**Published:** 2020-11-01

**Authors:** Arnab Kumar Mallik, Soumalya Mukherjee, Mahesh V. Panchagnula

**Affiliations:** 1 Department of Applied Mechanics, Indian Institute of Technology Madras, Chennai 600036, India; 2 Department of Biotechnology, Indian Institute of Technology Madras, Chennai 600036, India

## Abstract

The transport and deposition of micrometer-sized particles in the lung is the primary mechanism for the spread of aerosol borne diseases such as corona virus disease-19 (COVID-19). Considering the current situation, modeling the transport and deposition of drops in human lung bronchioles is of utmost importance to determine their consequences on human health. The current study reports experimental observations on deposition in micro-capillaries, representing distal lung bronchioles, over a wide range of *Re* that imitates the particle dynamics in the entire lung. The experiment investigated deposition in tubes of diameter ranging from 0.3 mm to 2 mm and over a wide range of Reynolds number (10^−2^ ⩽ *Re* ⩽ 10^3^). The range of the tube diameter and *Re* used in this study is motivated by the dimensions of lung airways and typical breathing flow rates. The aerosol fluid was loaded with boron doped carbon quantum dots as fluorophores. An aerosol plume was generated from this mixture fluid using an ultrasonic nebulizer, producing droplets with 6.5 *µ*m as a mean diameter and over a narrow distribution of sizes. The amount of aerosol deposited on the tube walls was measured using a spectrofluorometer. The experimental results show that dimensionless deposition (*δ*) varies inversely with the bronchiole aspect ratio ($\bar{L}$), with the effect of the Reynolds number (*Re*) being significant only at low $\bar{L}$. *δ* also increased with increasing dimensionless bronchiole diameter ($\bar{D}$), but it is invariant with the particle size based Reynolds number. We show that $\delta \bar{L}∼Re^{-2}$ for 10^−2^ ⩽ *Re* ⩽ 1, which is typical of a diffusion dominated regime. For *Re* ⩾ 1, in the impaction dominated regime, $\delta \bar{L}$ is shown to be independent of *Re*. We also show a crossover regime where sedimentation becomes important. The experimental results conclude that lower breathing frequency and higher breath hold time could significantly increase the chances of getting infected with COVID-19 in crowded places.

## I. INTRODUCTION

Several infectious respiratory diseases including corona virus disease-19 (COVID-19), threatening human lives globally, transmit primarily via virus laden droplets. Dramatic respiratory events such as coughs and sneezes that yield a large quantity of poly-dispersed droplets^1^ play a vital role in aiding such transmission.^2–6^ The respiratory exhaled liquid drops of an infected person can get into the respiratory tract of a healthy human and thus transmit the virus.^7–9^ The current outbreak of corona virus disease calls for the immediate attention of the research community as it demonstrates the global burden of severe respiratory diseases. An evocative understanding of the transport of these virus laden droplets inside the lungs is necessary to estimate the propensity of the virus and to treat it with pulmonary drugs.^10,11^ Thus, a study on dynamics of micrometer-sized droplets through micro-channels mimicking the lung environment finds relevance in order to estimate the deposition of virus laden drops in healthy human lungs. From a more fundamental perspective, aerosol deposition is enabled by a complex mix of several physical mechanisms such as impaction, diffusion, and sedimentation. The activity level of each physical mechanism is a strong function of the flow parameters including Reynolds (*Re*) and Stokes numbers. The Reynolds number, in particular, is important in understanding aerosol dynamics as it determines the relative importance of inertial effects to momentum diffusive effects. When *Re* is small, particle deposition is enabled primarily by diffusion toward the wall. In contrast, when *Re* is large, particle deposition is primarily known to occur by impaction. Therefore, it is important to develop a fundamental understanding of aerosol deposition as a function of the Reynolds number.

**FIG. 1.**
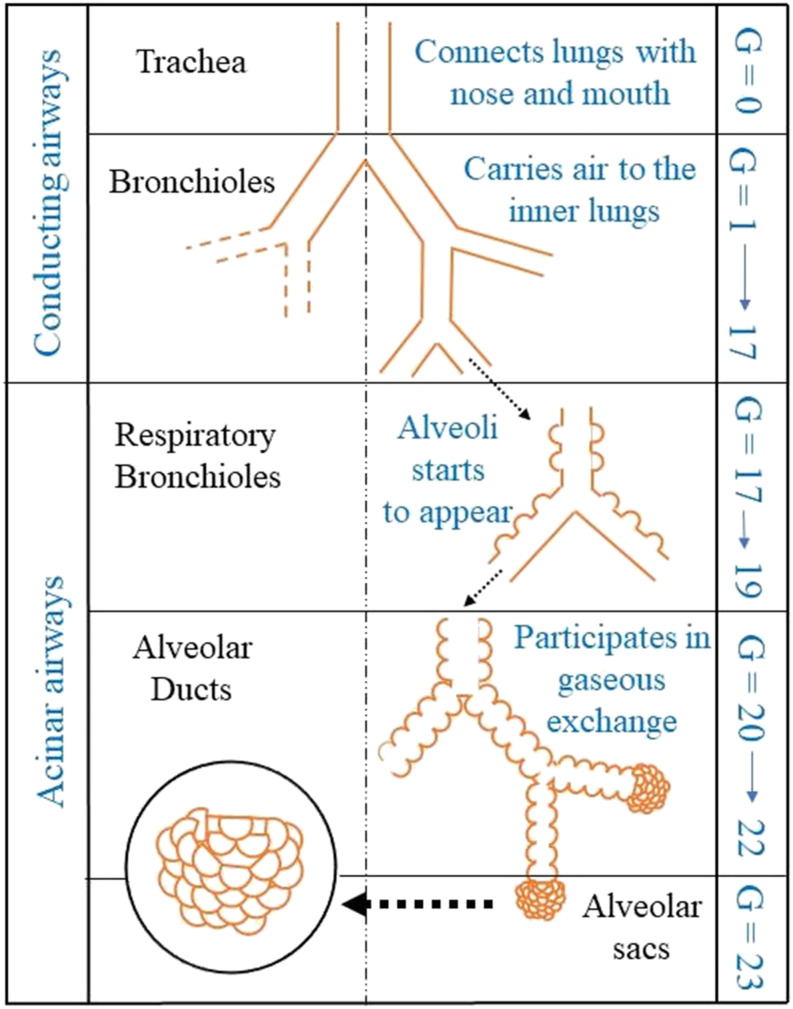
A schematic of the human lung morphology as proposed by Weibel^12^ showing the conducting and acinar airways. The conducting airways transport air from the nose and mouth to the lung through the trachea, whereas the acinar airways consisting of alveolar ducts participate in gas exchange with the blood.

The architecture of the human lungs consists of a branching network with sequences of bifurcation, known as bronchi. The diameter and length of the bronchi change at each bifurcation level, known as generations (*G*), starting with the trachea (*G* = 0). A quantitative morphological structure of the lung was proposed by Weibel,^12^ as shown in Fig. 1. The airways in the tracheobronchial region, i.e., 0 ⩽ *G* ⩽ 16, only conduct the flow of gases into and out of the lung. In the pulmonary region, i.e., 17 ⩽ *G* ⩽ 19, the air sacs known as alveoli appear on the wall of the airways, which are respiratory bronchioles. They are facilitated with the capillary blood supply that can exchange gases between the inhaled air and blood. For generations 20 ⩽ *G* ⩽ 23, the airways are completely made up of alveoli where *G* = 23 consists of clusters of alveoli participating in gaseous exchange. The length and diameter of the airways of the different generations of branching, starting from the trachea to the terminal alveolar sacs, vary over several orders of magnitude. The trachea has a diameter of 18 mm, $∼O\left(10^{1}\right)\text{mm}$, whereas *G* = 23 ends with a diameter of ≈0.41 mm, $O\left(10^{-1}\right)\text{mm}$.^12^ This variation of airway diameter of three orders of magnitude is responsible for a corresponding variation of the Reynolds number (*Re*) over the different generations.^13^ The Reynolds number in each generation is calculated based on the local bronchiole diameter and flow velocity. For the purpose of our experiments with phantom bronchioles, *Re* is calculated based on the micro-capillary diameter and flow velocity. Table I presents this information for various generations, estimated for a tidal volume of 500 ml (for a healthy human) under normal breathing conditions. Under these conditions, *Re* is $∼O\left(10^{3}\right)$ in the trachea and $∼O\left(10^{-2}\right)$ in the respiratory bronchioles and the alveoli. One of the challenging aspects of modeling the respiratory process is that every breath involves transport over six orders of magnitude *Re*. Consequently, an accurate model of aerosol transport would have to be able to model turbulent as well as diffusive transport. While the primary purpose of the respiratory system is to enable gas exchange, accompanying parasitic transport of inhaled aerosol is a necessary burden. The deposition of microdroplets in the lung depends on several biological factors such as the lung morphology and breathing patterns,^14^ as well as droplet morphology.^15,16^ On a related note, transmission of virus laden droplets and optimal inhalation therapy demand knowledge of lung morphometry. For example, inter-subject variability in lung morphometry may cause variation in health risk factors.^17,18^ The complicated geometry of the lung coupled with the extremely inconsistent nature of airflow during respiration makes accurate estimation of droplet deposition in alveoli challenging.^19^

**TABLE I.** Comparison of the bronchiole diameter with the branching generation in the lung. Typical *Re* is calculated based on regional airflow rates in the lung for a tidal volume of 500 ml and a 4 s breathing cycle.

Typical bronchiole diameter (mm)	Lung generation	Typical *Re*
10	0 → 6	10^3^–10^2^
1.5	7 → 10	40
1	11 → 13	5
0.5	14 → 21	10^−1^–10^−2^
0.3	22 → 23	7 × 10^−3^

Liquid particles are deposited in the lung through three mechanisms—*impaction, sedimentation,* and *diffusion*—depending on the local Reynolds number (*Re*).^20^ When *Re* is high or the aerodynamic size of the particle is large,^21^ the particles do not follow the airflow due to their inertia and impact the airway walls. This mode of deposition is called impaction. Since the momentum of the particles is a key factor, it is expected that this mode scales with the particle phase momentum flux. Impaction is responsible for deposition mainly in the throat, the trachea, and the upper generations of the lung.^22–27^ 10 ⩽ *Re* < 10^3^ is usually accompanied by intense activity when the breathing rate is high.^28,29^ Deposition by sedimentation typically occurs where the flow velocity is low, implying 1 ⩽ *Re* < 10, when gravitational force dominates over particle inertia.^30,31^ An increase in particle residence time in the airways, for example, during breath holding episodes, enhances the effect of this mechanism. Diffusion is a mechanism that is active at very low advection flow rates.^14,32^ For 10^−2^ ⩽ *Re* ⩽ 1 the diffusion becomes active, and is most active for small particle sizes. In this mechanism, Brownian motion causes the particles to move toward the wall. The particle flux to the wall is expected to scale as the inverse of the particle size and should be independent of the flow parameters since this mode is active when flow effects are suppressed. This type of deposition is noticed in the last few generations of the lung where the airway passages are extremely small, resulting in negligible flow velocity.^33^ It must be stated that aerosol deposition studies relevant to the end generations are rare in the current literature^34–36^ due to their microdimensions along with complex three-dimensional geometry. Fishler *et al.*^37^ reported experiments on a true scale acinar model with breathing and showed good agreement with numerical simulations. However, their study was restricted to constant diameter bifurcations. The investigation of Lin *et al.*^38^ with bifurcated bronchioles of differing diameter and low *Re* (between 0.1 and 1) was also restricted to a few generations of the lower lung. In order to gain a complete understanding of the deposition process, one needs to study the process over a range of Reynolds numbers where all three processes are allowed to dominate the deposition process.^72–77^ This forms the core motivation of this work—to develop an empirically initiated understanding of the droplet deposition process over a wide range of *Re* (typically five orders of magnitude), which will help us to understand the complete deposition mechanism in the entire lung, which is critical to mathematical modeling.^39,78–80^

The current work presents a fundamental view of droplet deposition on the wall of the bronchioles and mainly focuses on the transition of the deposition mechanism with several deposition parameters. The literature pertaining to aerosol deposition in circular tubes and channels^40^ is quite mature but at the same time mostly focused on high *Re* turbulent flows or laminar flows (*Re* ∼ 10^1^ − 10^2^) with higher diameter tubes.^41–49^ In all the cases, the deposition was characterized in a range of *Re* where impaction dominates the deposition process. The lacuna that the current work attempts to fill is to span a wider range of *Re* to gain a holistic understanding of aerosol deposition of all the underlying physical processes. The overarching goal is to construct a model that will be able to predict deposition in the entire lung. Toward this end, we will work with micro-capillaries (as phantom bronchioles) of varying diameters, mimicking the lung environment (see Table I for details) and subjecting the bronchioles to micrometer-sized droplet laden flows over a wide range of *Re* (10^−2^ ⩽ *Re* ⩽ 10^3^). This range represents airflow conditions in the entire lung from the trachea [$Re∼O\left(10^{3}\right)$] to alveoli [$Re∼O\left(10^{-2}\right)$]. Our analysis also points to a minimal set of dimensionless variables that are sufficient to capture the deposition physics over this wide range of *Re*. Finally, we will present an experimentally derived epidemiological model for regional deposition as a function of aerosol and flow parameters and henceforth estimate the regional deposition in lungs for different breathing rates and breath hold time to understand the adverse effect of virus laden drops.

## II. EXPERIMENTAL METHOD

### A. Experimental setup

**FIG. 2.**
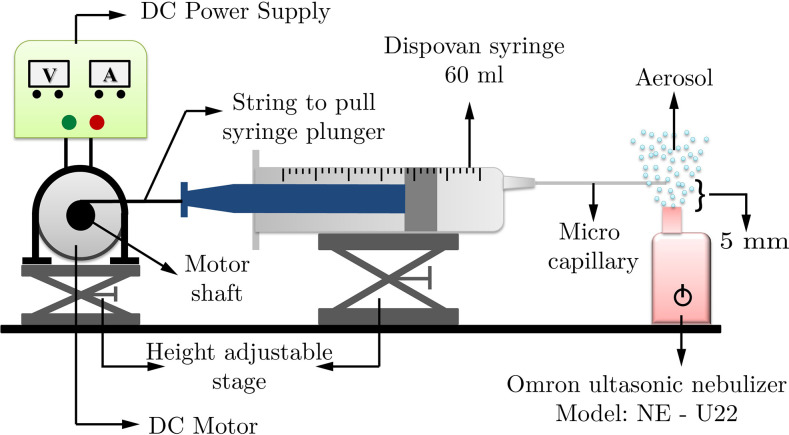
Schematic of the experimental setup consisting of an aerosol generator, micro-capillary, and syringe (60 ml capacity) with a motor arrangement to draw the syringe at a constant velocity. The inlet of the phantom bronchiole is placed at a height of 5 mm above the geometric center of the nebulizer exit. Different flow conditions through the micro-capillaries were achieved by controlling the rotational speed of the 12 V DC motor using a 32 V, 2 A DC power supply.

The experimental setup shown in Fig. 2 consists of a horizontal micro-capillary attached to a syringe of 60 ml. The deposition for different angles of inclination can be calculated from here by multiplying with the cosine as pointed out by Goldberg.^50^ Different diameters and lengths of these phantom bronchioles are intended to generate flows at different Reynolds numbers. Polytetrafluoroethylene (PTFE) tubules were used as the micro-capillary specimens for the deposition experiments. These tubules are translucent, are chemically inert, have low permeability, and have one of the lowest coefficients of friction of any solid. The non-sticky nature of this material helps in complete removal of the deposited aerosol by washing with water. PTFE tubules of different diameters, 0.3 mm, 0.5 mm, 1 mm, 1.5 mm, and 2 mm, were obtained from Cole–Parmer®. A syringe plunger intended to drive the flow is connected to the shaft of a DC motor. A DC motor of ratings 10 rpm at 12 V and 30 rpm at 12 V is used to achieve different suction rates. With the rotation of the motor shaft, the plunger is actuated at a constant rate to draw the aerosol exiting the nebulizer into the PTFE tubule. The rotational speed of the motor is varied to obtain different flow rates and thereby different Reynolds numbers. The rotational speed is, in turn, varied by varying the voltage at the motor terminals with the help of a 32 V, 2 A, DC power supply. For very low suction rates (*Re* ≪ 1), a syringe pump is used. Interestingly, the lowest *Re* experiment involved an ultra-low flow rate that required that a single experiment be performed carefully over a duration of 5 h in an ultra-quiet environment for reliable measurements. An ultrasonic mesh type nebulizer (Omron Model: NE-U22) is used to generate a finely atomized, gently rising aerosol plume. For all the runs, the temperature was maintained at room temperature, which was controlled at ∼25 °C. The setup is mounted on a height adjustable stage to ensure proper motion of the plunger and to maintain the micro-tubule at a height of 5 mm from the nebulizer exit, where all drop size measurements were performed.

### B. Preparation of aerosol liquid

The aerosol liquid composed of water as the fluid medium was loaded with boron quantum dots, which acted as fluorophores.^81^ The photo-physical properties of quantum dots, including carbon quantum dots (CDs), depend on their size^51^ as their bandgap originates from quantum confinement. The tuning of the bandgap, which is measured by the quantum yield (QY), can be achieved by incorporating trap sites while introducing functional groups during the synthesis of CDs. In order to increase the QY, trap sites of CDs are often doped with heteroatoms such as nitrogen,^52^ boron,^53^ or phosphorous^54^ depending on the application.

**FIG. 3.**
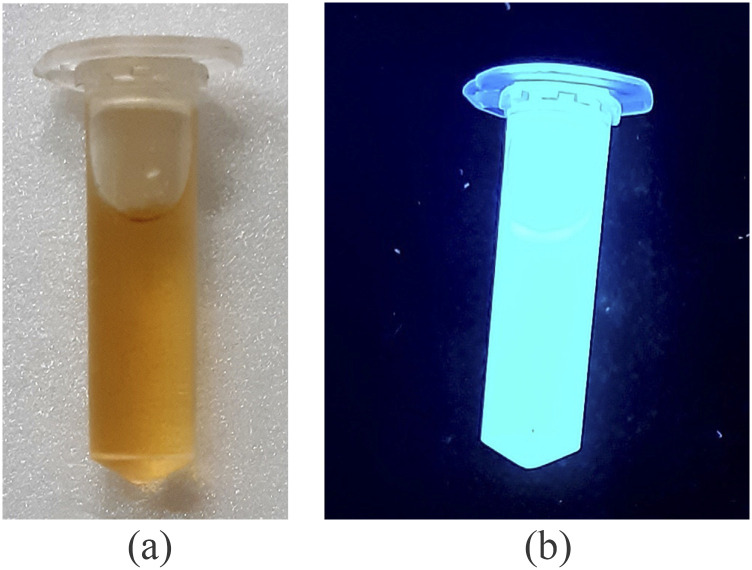
Images of vials (a) containing freshly prepared Boron doped Carbon Dots (BCDs) and (b) showing emission of blue light by BCDs when excited by UV light of 350 nm wavelength.

**FIG. 4.**
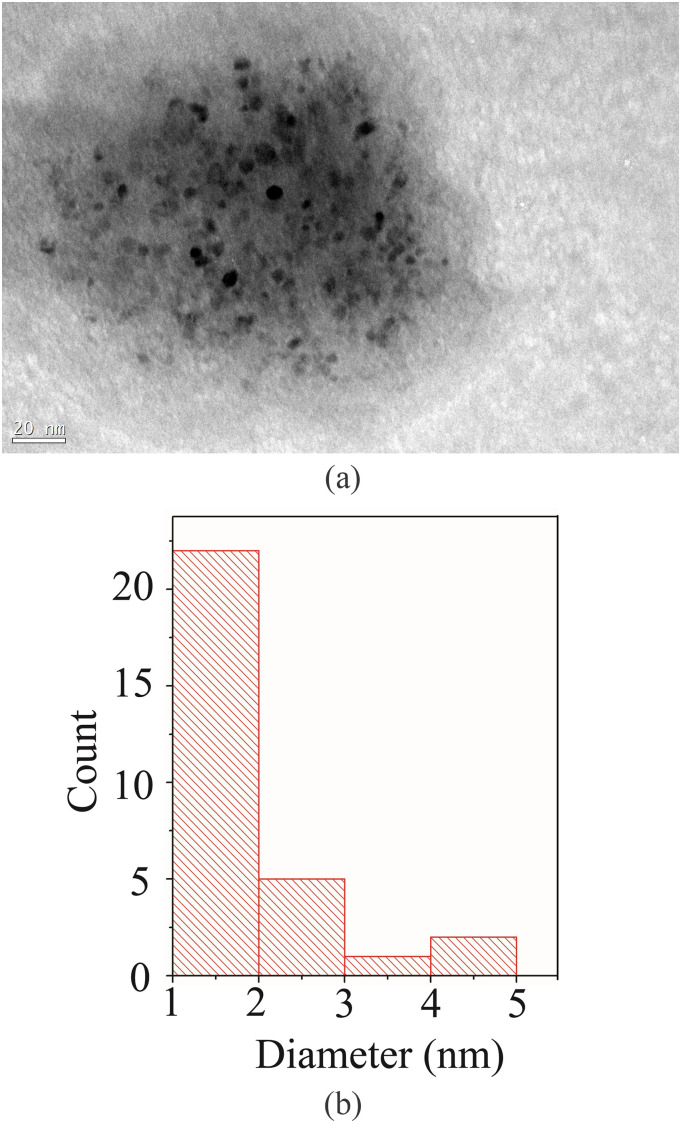
(a) Transmission Electron Microscopy (TEM) image of Boron Quantum Dots (BCDs). (b) Histogram of the size distribution of BCDs estimated from the TEM images. The BCD distribution is nearly monodisperse.

The synthesis of Boron doped Carbon Quantum Dots (BCDs) was done in the laboratory through a bottom-up process. Care was taken to ensure that the de-ionized (DI) water used for synthesis of CDs and BCDs has a pH of 7. All the chemicals used for synthesis were sourced from Sigma-Aldrich®, Merck®, Fischer Scientific®, and Spectrochem®. Equimolar concentration solutions at 0.5M each of boric acid (boron precursor) and glucose (carbon precursor) were prepared with 10 ml of DI water and mixed using a magnetic stirrer at 300 rpm for 15 min. The solution was transferred to a glass bowl and treated under commercial microwave (IFB®) radiation of 700 W for 5 min. The resulting solid was then dried in vacuum to remove all volatiles and dispersed in 500 ml of DI water to form an olive green solution, as shown in Fig. 3(a). The larger particles were sorted out by centrifugation at 1500 g for 15 min and filtered in vacuum using a 10 kDa filter. The resultant solution emits blue light when excited by a wavelength of 350 nm, as shown in Fig. 3(b). The morphological characteristics of BCDs were investigated by Transmission Electron Microscopy (TEM) [see Fig. 4(b) for a representative image]. Their size distribution was estimated from the TEM images with the help of ImageJ® software. Figure 4(b) shows the obtained size distribution. As can be seen, the BCDs are nearly monodisperse and range in size from 1 nm to 2 nm [Fig. 4(b)], which is ∼5 orders smaller than the smallest dimension of the phantom bronchiole used in our experiment. Since the BCDs were nearly mono-disperse, the emitted fluorescence spectrum is likely to be in a narrow wavelength band.

### C. Characterization of aerosol plume

The respiratory events generate a wide range of droplets based on cough, sneeze, speech, and breath.^55^ The study of Duguid^56,57^ showed that 95% of the drops generated during sneeze and cough lie between 2 *µ*m and 100 *µ*m. According to Yang *et al.*,^58^ the drop size ranges between 0.62 *µ*m and 15.9 *µ*m during cough with a mean drop size of 8.35 *µ*m. The droplet generated due to breath is very small, ranging between 0.15 *µ*m and 0.19 *µ*m,^59,60^ which can easily travel to the deep lung, causing enormous health impact. In this study, a mesh type ultrasonic nebulizer was used to generate the poly-dispersed aerosol plume. The nebulization flow rate was maintained constant at 0.25 ml/min. The droplet size and velocity distributions of the aerosol plume generated by the nebulizer were characterized using a TSI® Phase Doppler Particle Analyzer (PDPA). The PDPA is a non-intrusive, laser-based, single particle and point measurement system that works on the principle of interferometric particle sizing. The optical settings employed for the PDPA are given in Table II. Since accurate size measurement depends on the phase difference of photodetectors, phase calibration was periodically performed to avoid unexpected phase delay. The optical settings of the PDPA were adjusted such that the particle diameter measurement range is 0.5 *µ*m–165 *µ*m with an estimated accuracy of ±0.1 *µ*m over the entire range. A wide range of velocity measurements from −100 m/s to 200 m/s was also possible through the appropriate bandpass filter choice. As a result, drop size and velocity were measured with an accuracy of ±0.2%. In addition, the photomultiplier tube (PMT) voltage is chosen such that it does not add noise to the data while producing a good data rate. Finally, care was taken to ensure that the validation rate was always greater than 95%. This ensured that the drop size distribution was measured with a high degree of fidelity.^61^

**TABLE II.** Optical settings of the PDPA.

Optical settings	Values
Transmitter wavelength	532 nm
Transmitter focal length	363 mm
Laser beam separation	50 mm
Laser beam diameter	2.10 mm
Beam expander ratio	1
Beam waist	117.09 *µ*m
Fringe spacing	3.8715 *µ*m
Bragg cell frequency	40 MHz
Off-axis angle	43°
Mode of scattering	Refraction
Refraction index	1.33

The diameter and velocity of the aerosol plume exiting the nebulizer are measured at different radial locations, 2 mm apart, at an axial distance of 5 mm from the nebulizer exit. For ensuring high statistical reliability of the PDPA measurement, 10 000 drops were sampled at each measurement location. The PDPA only yields point-wise drop size distribution data. A global size and velocity distribution, characteristic of the entire nebulizer, was calculated from the point-wise data following the method of Tratnig and Brenn^62^ and Dhivyaraja *et al.*^63^ The global probability density functions (Pdfs) of both drop size and velocity are true representations of nebulizer performance since they are insensitive to external factors.^64^

**FIG. 5.**
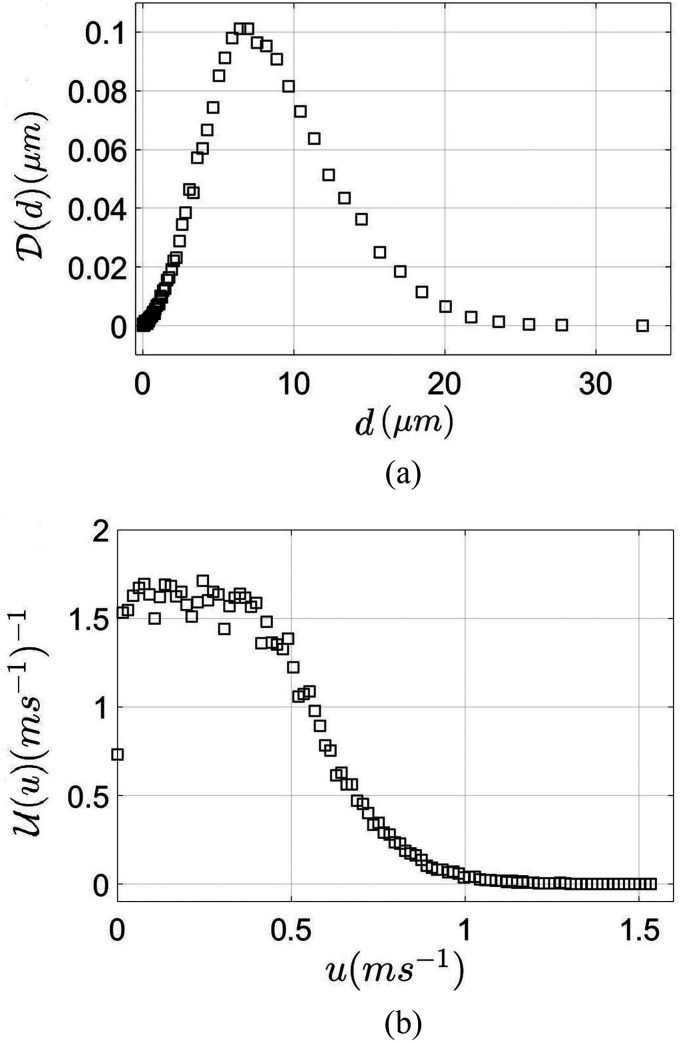
Plot of (a) the global diameter probability distribution function and (b) the global velocity probability distribution function, both measured using the PDPA.

The global aerosol drop size pdf [D(d)] is shown in Fig. 5(a). The size distribution of the droplets represents a smaller range of drop sizes generated from cough and sneeze. However, the distribution exactly represents the drop sizes generated during loud speech.^65^ The mode of the distribution is at 6.5 *µ*m, which is taken as the characteristic droplet size in the aerosol plume. The velocity pdf [U(u)] in Fig. 5(b) denotes that the most probable velocity occurs for 0 < *u*(m/s) < 0.5. The mean velocity in this range from this pdf was found to be 0.44 m/s. We would like to ascertain that coagulation is not significant, especially in the longer duration experiment. The granular temperature of the rising plume measured as the variance of the velocity pdf is an important measure of the collision frequency, which could in turn lead to coagulation. From the measured velocity pdf, it was estimated that only 11% of the total kinetic energy was contained in disorderly motion as granular temperature; the remaining 89% was contained in the mean motion of the droplets. This kinetic energy of the droplets in the disordered motion is further damped when the droplet laden gas enters the test micro-capillary environment due to the lower *Re* in the capillary in comparison to that of the plume. Therefore, it was estimated that the droplet collision frequency leading to droplet coagulation is further reduced and negligible. The deposition fraction is measured in each experiment as the ratio of the deposited aerosol to the amount of aerosol that is present in the volume of air drawn into the capillary representing the distal bronchiole. For this purpose, the aerosol volume fraction (*α*_0_) is estimated. This parameter can be estimated from the PDPA-measured particle concentration (which is equal to 9.3 × 10^4^*cc*^−1^ on the centerline from where the plume is drawn into the test section). By multiplying the particle concentration with a representative drop volume, *α*_0_ can be estimated to be 6.26 × 10^−5^. Since the volume fraction is ∼10^−5^, the aerosol plume can be construed to be dilute and consist of non-interacting droplets. From these measurements, one can conclude that a finely atomized, gently rising, sparse non-coagulating aerosol plume was formed by the nebulizer.

Since the aerosol size distribution formed by the nebulizer is poly-dispersed, it is important to ascertain that the deposition process for drops of all sizes in the range of particle sizes at any operating condition is dominated by a single physical process.^66^ For this purpose, we have computed the range of Stokes numbers (Stk) and Schmidt numbers (Sc) associated with the complete range of drop sizes in the plume. The Stokes number is given by $Stk=\frac{\rho_{p}d_{p}^{2}u_{0}}{18\mu_{g}l_{0}}$, where *ρ*_*p*_ is the particle density, *d*_*p*_ is the particle diameter, *μ*_*g*_ is the gas medium viscosity, *u*_0_ is the free stream velocity, and *l*_0_ is the characteristic length. The Schmidt number is given by $Sc=\frac{\mu }{\rho D}$, where *μ* is the fluid viscosity, *ρ* is the fluid density, and *D* is the mass diffusivity. Table III presents a listing of the ranges of these numbers for various operating *Re* conditions. As can be seen, the Stokes number associated with the smallest as well as the largest aerosol particle in the distribution was never greater than 10^−3^ for all the experiments reported herein. In addition, the Schmidt number (based on particle diffusivity) for the minimum as well as maximum size particles in the plume was at least 10^5^ for all cases of *Re*. In other words, the particle deposition for all the particles in the distribution was characterized by low Stokes and high Schmidt numbers. This fact further reinforces the idea that the deposition physics associated with particles in the entire distribution is similar—one that faithfully follows the flow field and one that is diffusion dominated. The deposition characteristics are, therefore, a function of *Re* only and not influenced by the fact that the aerosol plume is not monodisperse.

**TABLE III.** A table listing the range of Stokes and Schmidt numbers for all size drops in the complete set of experiments. *d*_*min*_ = 1 *µ*m and *d*_*max*_ = 20 *µ*m from the measured drop size *pdf*.

*Re*	Stokes number	Schmidt number
10^3^	4.70 × 10^−11^ → 1.90 × 10^−8^	6.10 × 10^5^ → 1.20 × 10^7^
10^2^	4.70 × 10^−10^ → 1.90 × 10^−7^	6.10 × 10^5^ → 1.20 × 10^7^
10	4.70 × 10^−9^ → 1.90 × 10^−6^	6.10 × 10^5^ → 1.20 × 10^7^
1	4.70 × 10^−8^ → 1.90 × 10^−5^	6.10 × 10^5^ → 1.20 × 10^7^
10^−1^	4.70 × 10^−7^ → 1.90 × 10^−4^	6.10 × 10^5^ → 1.20 × 10^7^
10^−2^	4.70 × 10^−6^ → 1.90 × 10^−3^	6.10 × 10^5^ → 1.20 × 10^7^

### D. Preparation and analysis of deposition samples

The fluorescence signal from the BCDs in the sample was measured using a Horiba FluoroMax® spectrofluorometer. The instrument consists of a 150 W xenon arc lamp, which were self-calibrated for all wavelength drives and slits. The fluorescence detector consisted of a photomultiplier tube (PMT) that can capture emission wavelengths from 185 nm to 850 nm with an accuracy of ±0.5 nm and a repeatability of 0.1 nm. The water Raman signal to noise ratio was found to be 6000:1, as calculated using the First Standard Deviation (FSD) method, and 16 000:1, as calculated using the root mean square (rms) method. This ensured that the fluorescence signal from the BCDs was not confounded by the signal from other sources.

The experiment involved an aerosol plume flowing through the PTFE micro-capillaries at a prescribed flow rate. The deposited aerosols within the capillary were then flushed thoroughly with 6 ml of DI water to prepare the samples for measuring aerosol concentration. From this, a sample volume of 2 ml of the solution is taken in the cuvette and excited with the wavelength of 340 nm with a slit width of 3 nm. The emission intensity was recorded for different wavelengths varying from 405 nm to 610 nm, with a slit width of 5 nm. The sensitivity of the instrument was checked for an empty cuvette, a cuvette containing DI water, as well as a cuvette containing fluorescence samples for the lowest deposition recorded. It was found that the intensity of the water containing a fluorescence sample is at least one order higher than that of normal DI water. The intensity obtained for various aerosol deposition measurements is compared with the intensity obtained from 1 ml of aerosol liquid (i.e., BCD) dissolved in the same volume of DI water used for flushing the capillaries. This is taken as the reference value for estimating the deposition in the distal bronchiole. Finally, the concentration of the deposited aerosol was calculated from the fluorescence measurement.

## III. RESULTS

The deposition of microdroplets is investigated for micro-capillaries of differing lengths and diameters to mimic flow at different Reynolds numbers. The deposition concentration of aerosol in these distal bronchioles can be expressed as a function of several parameters as

$$d=f\left(L,D,Q,T,\nu ,D_{10},\alpha_{0}\right).$$ (1)

Here, *d* is the measured droplet deposition (ml), *α*_0_ is the volume fraction of the drop from the nebulizer (ml of aerosol per ml of space), *L* is the length of distal bronchioles (mm), *D* is the distal bronchiole diameter (mm), *Q* is the volume flow rate (ml/s), *T* is the time duration of the flow (s), *ν* is the kinematic viscosity of air (m^2^/s), and *D*_10_ is the mean droplet diameter (*μ*m). From a careful dimensionless analysis, one can identify the following relevant dimensionless parameters from the above parameters using the Buckingham Π theorem. These dimensionless parameters are defined in Table IV.

**TABLE IV.** Definition of dimensionless parameters.

Dimensionless parameter	Definition
Deposition fraction (*D*_*F*_)	*d*/(*α*_0_*QT*)
Aspect ratio ($\bar{L}$)	*L*/*D*
Dimensionless tubule diameter ($\bar{D}$)	*D*/*D*_10_
Reynolds number (*Re*)	$4Q/\left(\pi \nu D\right)$
Dimensionless time ($\bar{T}$)	$4QT/\left(\pi D^{3}\right)$

The deposition of droplets is investigated over a wide range of flow conditions, 10^−2^ ⩽ *Re* ⩽ 10^3^. If *QT* is the total volume of air that has flown through the distal bronchioles during the experiment, *α*_0_*QT* is the total volume of aerosol that entered the distal bronchioles as a result. The deposition fraction (*D*_*F*_) is the fraction of the exposed aerosol (*α*_0_*QT*) that has been deposited in the distal bronchioles. This is likely to increase with increasing length of the bronchiole and time of exposure.^31^ In order to normalize for these effects, a dimensionless deposition fraction per unit (dimensionless) length and (dimensionless) time, *δ* is defined as

$$\delta =\frac{D_{F}}{\bar{L}\bar{T}}.$$ (2)

Equation (1) can be rewritten in terms of the dimensionless parameters in Table IV as

$$\delta =G\left(Re,\bar{D},\bar{L},\bar{T},\alpha_{0}\right).$$ (3)

It is the goal of this work to identify a physics-consistent and universal function *G* as in Eq. (3) from experimental data.

The deposition experiments were repeated several times for different flow conditions to ascertain the repeatability of the deposition fraction in the distal bronchioles. The maximum estimated uncertainty^67^ for all the measured parameters was within ±5%. The values are given in Table V. We will now investigate the effect of the various dimensionless groups on the dimensionless deposition, *D*_*F*_ and *δ*.

**TABLE V.** Uncertainty in the measured and calculated parameters.

Derived parameters	Estimated uncertainty (%)
Deposition fraction (*D*_*F*_)	±5
Aspect ratio ($\bar{L}$)	±1
Dimensionless diameter ($\bar{D}$)	±1.5
Reynolds number (*Re*)	±0.2
Dimensionless time ($\bar{T}$)	±0.6
*Δ*	±5

### A. Effect of $\bar{L}$, $\bar{D}$, and *Re* on deposition

**FIG. 6.**
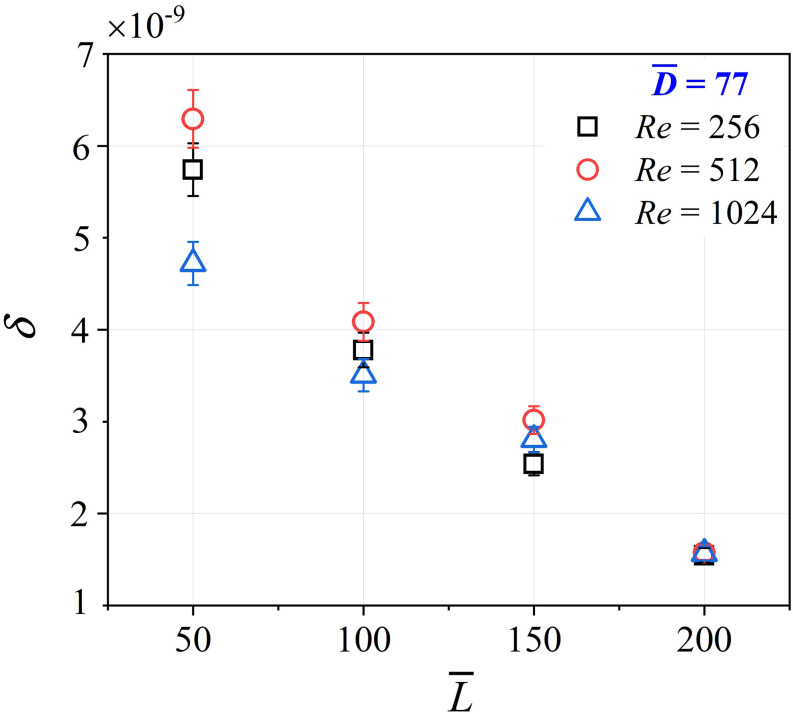
Plot of the variation of dimensionless deposition (*δ*) vs aspect ratio ($\bar{L}$) for different Reynolds numbers (*Re*). The data show an inverse relation of *δ* with the bronchiole aspect ratio ($\bar{L}$). For high $\bar{L}$, the effect of *Re* is negligible.

Figure 6 presents the variation of *δ* with $\bar{L}$ for different values of *Re*. The values of *Re* are chosen such that they span the range of Reynolds numbers encountered in the upper bronchi, where inertial effects are significant. $\bar{D}$ was maintained constant at 77 for all data in Fig. 6. The results show that *δ* decreases with an increase in $\bar{L}$. In other words, the deposition per unit length and unit time decreases as the length of the tubule increases. For small values of $\bar{L}$, the effect of *Re* is visible. For small $\bar{L}$, inertial effects play a role in decreasing deposition. However, for high aspect ratios, the effect of *Re* is insignificant. It is also noted that for all the cases, the deposition is highest for *Re* = 512 and lower for both *Re* = 1024 and *Re* = 256. High *Re* produces high velocity (since the capillary diameter is constant), which causes the aerosol particles to pass through the capillary rather than depositing on the wall. On the other hand, low *Re* reduces the flow rate (since *Re* = *Q*/*νD*), simultaneously reducing the ingress of the particles in the tube. This may be a reason for *δ* showing non-monotonic variation with *Re*. Nevertheless, this is an interesting observation that deserves further investigation.

**FIG. 7.**
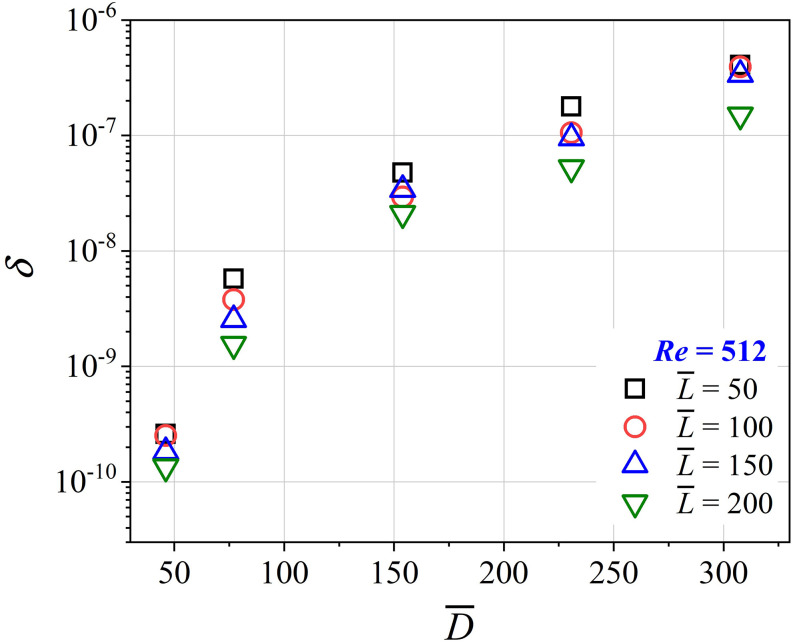
Plot of dimensionless deposition (*δ*) vs dimensionless bronchiole diameter ($\bar{D}$) for varying bronchiole aspect ratios ($\bar{L}$). The Reynolds number is constant at *Re* = 512. It is seen that with an increase in $\bar{D}$, the dimensionless deposition (*δ*) increases, while $\bar{L}$ still follows the inverse relation with *δ* for a constant $\bar{D}$.

**FIG. 8.**
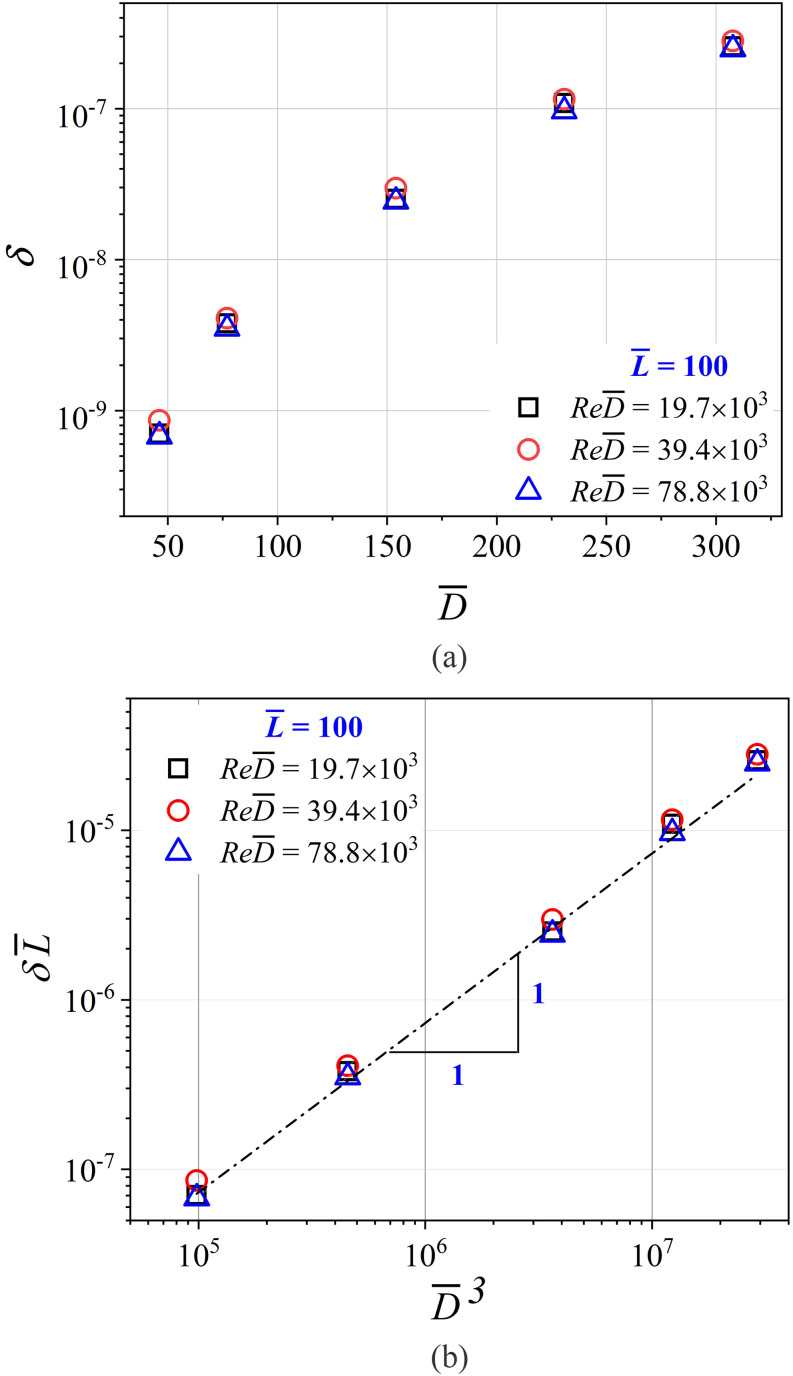
(a) Plot of dimensionless deposition (*δ*) vs $\bar{D}$ for varying particle-based Reynolds numbers ($Re_{p}=Re\bar{D}$). $\bar{L}=100$ for all data in this plot. It is seen that *δ* increases with $\bar{D}$. $Re\bar{D}$ does not affect the deposition. (b) The same data are re-plotted to show the variation of $\delta \bar{L}$ vs ${\bar{D}}^{3}$. The best fit power law is given by $\delta \bar{L}=7.3\times 10^{-13}{\bar{D}}^{3}$. The fit is independent of $Re\bar{D}$. *Re* ∼ 10^3^ for all data in these plots, implying impaction-dominated deposition.

The effect of the dimensionless bronchiole diameter on deposition for constant *Re* = 512 is shown in Fig. 7. *δ*, representing the deposition fraction per unit dimensionless length of the bronchiole per unit dimensionless suction time, increases with an increase in bronchiole diameter, represented by $\bar{D}$. The rate of increase in *δ* decreases for $\bar{D}>150$. This is because for a constant *Re*, the increase in bronchiole diameter requires the flow rate to increase since *Re* = *Q*/*νD*, which increases the deposition significantly. The value of *δ* is lowest for $\bar{L}=200$ and increases with a decrease in $\bar{L}$, similar to the trends shown in Fig. 8. Figure 8(a) is a plot of *δ* vs $\bar{D}$ for different flow conditions. The suction flow rates are represented by a particle-based Reynolds number *Re*_*p*_ = 4*Q*/(*πνD*_10_). Interestingly, $Re_{p}=Re\bar{D}$, which can be understood as a Reynolds number based on the mean particle size, *D*_10_. As can be seen, *δ* increases with an increase in the dimensionless bronchiole diameter ($\bar{D}$), but is not dependent on the particle-based Reynolds number. This is unlike the data presented for a constant *Re* in Fig. 7. As $\bar{D}$ increases at a constant $Re\bar{D}$, the mean velocity of the airflow in the bronchiole decreases. This causes the rate of deposition to increase since diffusion-driven and gravitational settling become relevant. It is important to note that both Figs. 7 and 8 are plotted with the *δ* coordinate being plotted on a logarithmic axis. A factor of 6 change in $\bar{D}$ brings about three orders of magnitude change to the deposition fraction. Therefore, it can be concluded that the bronchiole diameter is an important parameter in determining aerosol deposition. Figure 8(b) presents the same data as in Fig. 8(a) with the abscissa now changed to ${\bar{D}}^{3}$ and the ordinate re-scaled to $\delta \bar{L}$. Clearly, $\delta \bar{L}∼{\bar{D}}^{3}$ and is independent of *Re*. We will investigate this further in Sec. III B.

### B. Deposition for the entire *Re* range

Further experiments were carried out for different orders of magnitude of *Re* ranging from 10^−2^ ⩽ *Re* ⩽ 10^3^ in this study. To the best of our knowledge, this is the first report where the entire dynamic range of lung-relevant Reynolds numbers has been explored in one experiment.

**FIG. 9.**
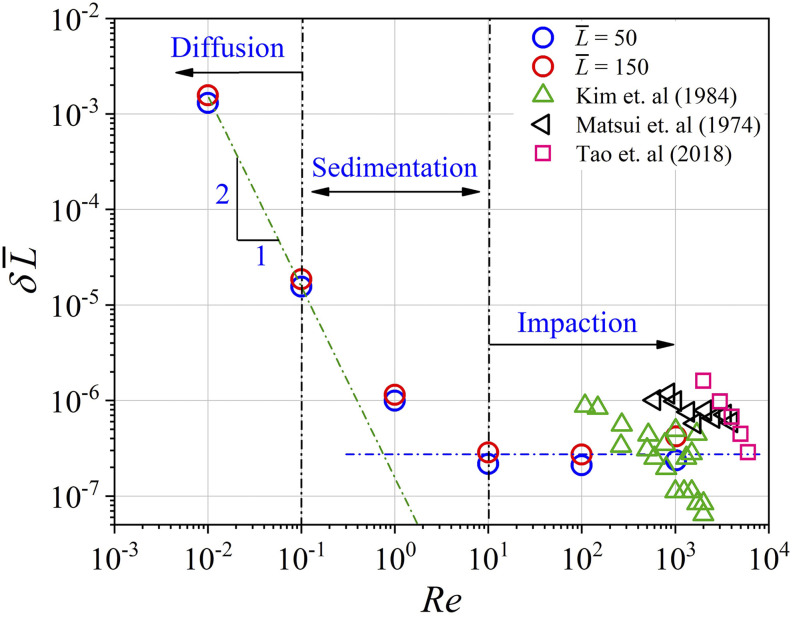
Plot of $\delta \bar{L}$ vs *Re* for $\bar{D}$ being constant at 77 and for $\bar{L}=50$ and 150. *Re* varies over five orders of magnitude. Interestingly, $\delta \bar{L}∼Re^{-2}$ for *Re* < 1 and is constant for *Re* > 1. In the plot, the green dashed-dotted line is the best fit for *Re* < 1 given by $\delta \bar{L}=1.55\times 10^{-7}Re^{-2}$. The blue dashed-dotted line is the best fit for *Re* > 1 given by $\delta \bar{L}=2.73\times 10^{-7}$. Figure legend: green triangles—Kim *et al.*;^47^ left pointing triangles—Matsui *et al.*;^68^ and pink squares—Tao *et al.*^69^

The effect of *Re* on $\delta \bar{L}$ is investigated for $\bar{L}=50$ and 150 in Fig. 9. It may be recalled that $\delta \bar{L}$ is the dimensionless rate of deposition. It can be seen that when $\delta \bar{L}$ is plotted against *Re*, the data in Fig. 9 nicely collapse onto a single curve. This data collapse points to a minimal set of dimensionless parameters that is required to completely describe aerosol deposition. Data from the literature have also been re-plotted in the current nomenclature in Fig. 9. First, it can be seen that at any value of *Re*, there is a significant variation in the data from the literature. Second, the data in the literature are limited to values of *Re* > 10^2^. It can be seen from Fig. 9 that our data are within the range of values from the literature. Two asymptotic regimes can be seen in Fig. 9. For *Re* > 1, $\delta \bar{L}$ is independent of *Re*. This is the parametric regime where deposition happens mostly due to impaction on the bronchiole walls. For *Re* < 1, $\delta \bar{L}∼Re^{-2}$. For *Re* = 1, the velocity of the flow is in $∼O\left(10^{-2}\right)\text{ m}/\text{s}$, which causes sedimentation of particles and increases *δ* by an order of magnitude. A further reduction in *Re* causes the suction velocity to be small enough that diffusion becomes the dominant mode of deposition. In addition, $\delta \bar{L}$ increases by several orders of magnitude as *Re* is decreased from 10^3^ to 10^−2^, i.e., from the impaction regime to the diffusion regime. The order of $\delta \bar{L}$ is almost constant in the impaction regime, while it increases for *Re* < 1.

## IV. DISCUSSION

The data collapse in Figs. 8(b) and 9 points to a universal description of deposition in dimensionless terms as a function of $\bar{D}$ and *Re*, the two abscissa parameters in these figures. A best fit of the data in Figs. 8(b) and 9 is given by

$$\delta \bar{L}=7.3\times 10^{-13}{\bar{D}}^{3}\text{ if}Re\gg 1$$ (4a)

$$=1.55\times 10^{-7}Re^{-2}\text{ if}Re\ll 1.$$ (4b)

Equations (4) explain the data in both Figs. 8 and 9. This is the explicit form of Eq. (3) that we set out to identify. Equations (4) also indicate the minimal set of dimensionless parameters that are required to model aerosol deposition over the entire range of operating conditions.

In order to study the physics underlying Eqs. (4), it is useful to recast Eqs. (4) back into the dimensional parameter form. From the dimensional form of Eq. (4a) for *Re* ≪ 1, we find that *d* ∝ *ν*^2^*T*^2^*α*_0_/*D*_10_. Recall that *d* is defined as the total volume of aerosol deposited in time *T*. For *Re* ≪ 1, as expected, the flow rate and other parameters do not play a role. The deposition depends linearly on the particle concentration *α*_0_. The rate of deposition is proportional to $D_{10}^{-1}$ as one would expect in diffusion-dominated deposition (since the diffusion co-efficient scales as $D_{10}^{-1}$). From the dimensional form of Eq. (4b) for *Re* ≫ 1, we find that $d\propto Q^{2}T^{2}\alpha_{0}/D_{10}^{3}$. Again, as expected, the deposition in this case is dependent on the square of the velocity (*Q*^2^) and depends linearly on the particle concentration (*α*_0_) since *d* is dominated by impaction. Therefore, we conclude that the empirically motivated correlations that we have presented are also consistent with the physics-based scaling laws in the respective regimes.

Equation (4) is a closed form expression to estimate the deposition in the lung bronchiole. We have ignored the effect of the lung orientation in this study. However, as pointed out by Goldberg and Smith,^50^ one could account for the orientation angle by re-scaling time using the cosine of the angle of inclination. In conclusion, we have developed a quantitative physics-consistent correlation to predict the rate of deposition in any bronchiole. This modeling approach is grounded in experiments and could be construed to be complementary to purely computational simulation^70^ approaches that are being pursued in the recent literature.

## V. ESTIMATION OF REGIONAL DEPOSITION FROM EXPERIMENTAL MODEL

The intention of developing the model in Eqs. (4) from the experimental results is to calculate the regional deposition in lungs for estimating the propensity of a virus infected droplet entering and depositing in the human lung. The virus laden droplets may start showing its harmful effects only after they reach the respiratory zone of the lungs where they come in contact with the blood stream.^32^ Therefore, an estimation of alveolar deposition for varying breathing frequency and breath hold time is important to understand the dynamics of infection. It is well known that the breathing frequency and breath hold time have a significant effect on aerosol deposition.

**FIG. 10.**
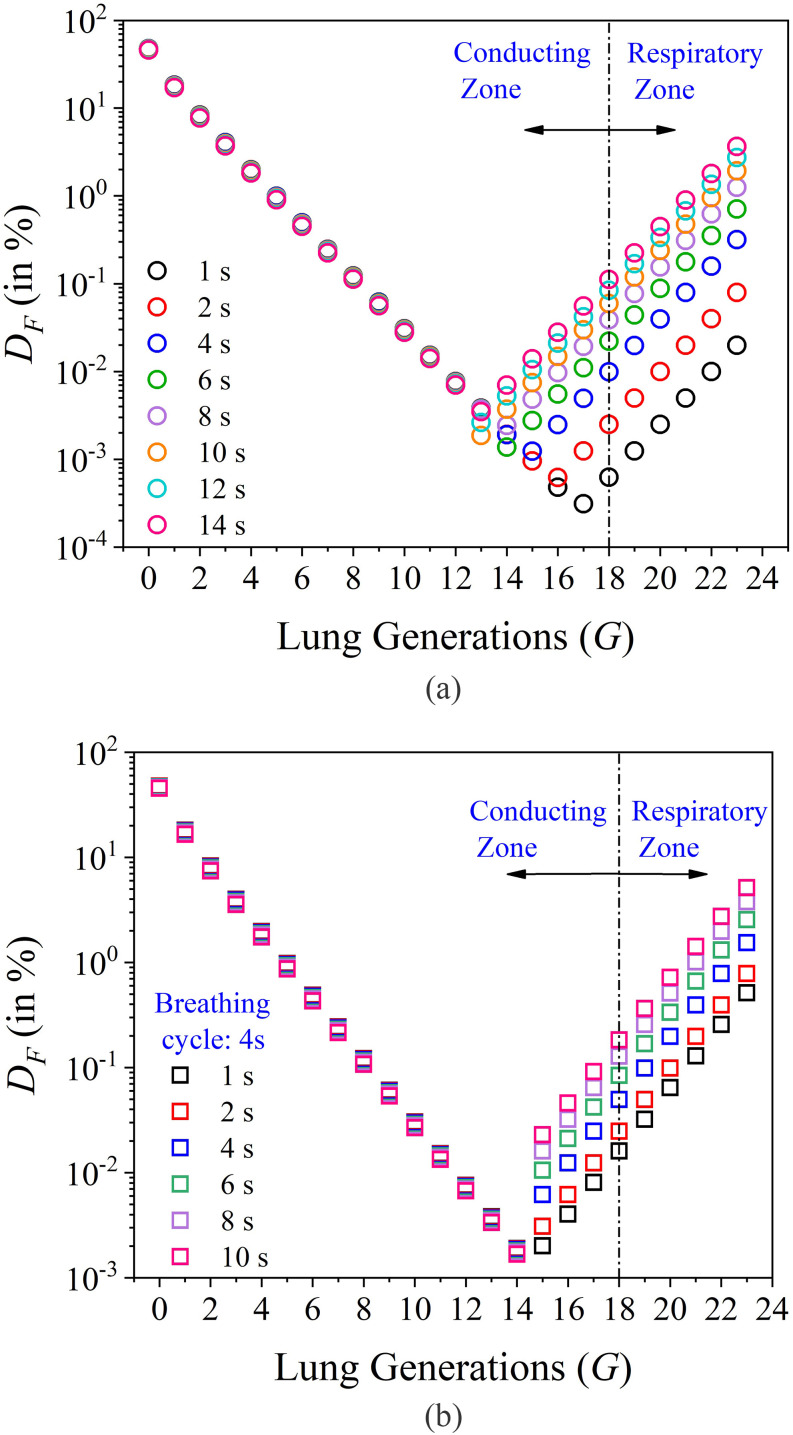
(a) Plot of *D*_*F*_ and lung generations (*G*) for different breathing frequencies. The time for each breathing cycle is considered as the time for inspiration and expiration together. (b) Plot of *D*_*F*_ and *G* for different breath hold time. For both the plots, it can be seen that the model for *Re* ≫ 1 does not show any response with breathing frequency and breath hold time, whereas the model for *Re* ≪ 1 is found to be very sensitive for both of these parameters.

The above developed model is used to analyze the effect of breathing frequency and breath hold time for the deposition of a particle sized ∼O(10 μm) (it must be mentioned that the final conclusions are independent of this choice). Figure 10(a) shows the effect of breathing frequency on regional deposition in lungs. The regional deposition fraction is analyzed for varying time periods of breathing (inspiration plus expiration) ranging from 1 s to 4 s. The variation of breathing frequency is mainly due to different types of activities we do throughout the day. The breathing cycle of 1 s (inspiration for 0.5 s and expiration for 0.5 s) takes place at the time of intense activity such as running, swimming, cycling, climbing, and other different workouts. The breathing cycle of 2 s represents moderate activities such as walking and light workouts, and the breathing cycle of 4 s represents sedentary activities such as sitting and lying down, which also represent the normal breathing cycle of a human adult. The regional deposition for the normal breathing cycle matches well with that of Hinds^71^ for particle size in the order of 10 *µ*m. It can be seen in Fig. 10 that the deposition decreases exponentially from 0 ⩽ *G* ⩽ 11 and rises exponentially for 12 ⩽ *G* ⩽ 23. It may be recalled that *G* denotes the dichotomous branching generation number in the lung. The deposition model in Eqs. (4) is valid in the asymptotic limits of *Re* ≪ 1 and *Re* ≫ 1. The region near *Re* ∼ 1 is a crossover region where the equations are strictly not valid. This is the source of the discontinuity in Fig. 10.

From Fig. 10(a), it can be seen that for intense activity, the deposition in the alveolar region is significantly low and the total deposition in lungs is decreased. With a decrease in breathing frequency, the deposition for *Re* < 1 increases, whereas the deposition for *Re* > 1 is constant. This is because the model for *Re* ≪ 1 [refer to Eqs. (4)] indicates that the dimensionless deposition is proportional to *Re*^−2^, whereas for *Re* ≫ 1, the deposition is independent of *Re*. Thus, lower breathing frequency increases deposition in the alveolar region of the lungs due to high residence time of the aerosol, which enhances the diffusion process. Thus, long breaths in crowded places may be a threat for virus infections.

**FIG. 11.**
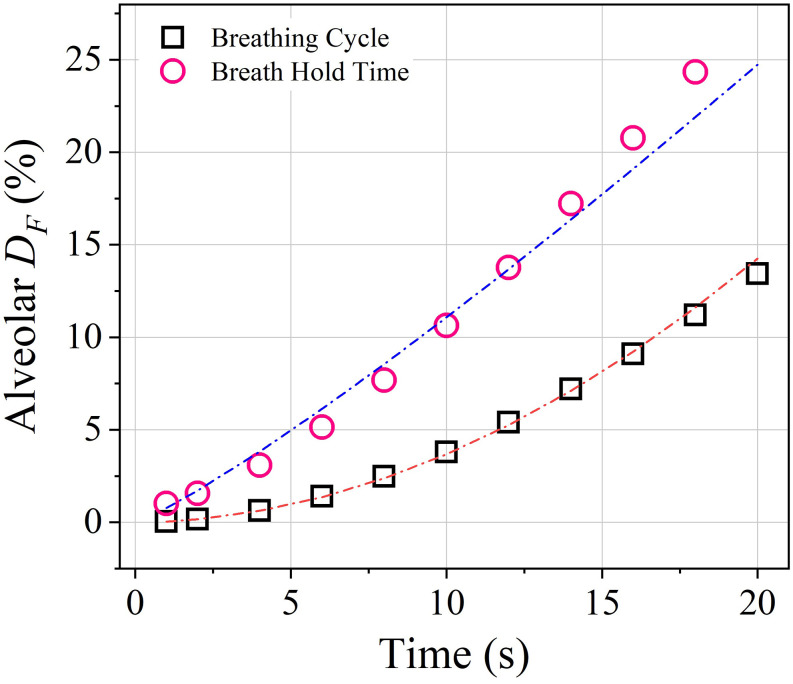
Plot of the variation of the alveolar deposition fraction (*D*_*F*_) with time for different breathing frequencies and breath hold time. The alveolar for different breathing cycles is of non-linear nature where *D*_*F*_ scales to *T*^2^ (blue dashed-dotted line: *D*_*F*_ = 0.04*T*^2^). The alveolar deposition for different breath hold time is somewhat linear where *D*_*F*_ scales to *T*^1.16^ (red dashed-dotted line: 0.766*T*^1.16^).

The alveolar deposition can be further increased by introducing a breath hold time between inspiration and expiration. Figure 10(b) shows the effect of the breath hold time on alveolar deposition for a breathing cycle of 4 s. The breath hold time contributes to the diffusion deposition process (*Re* < 1) that is dominant in the alveolar region, in turn increasing aerosol deposition in the alveolus. Figure 11 shows that the increase in alveolar deposition is non-linear with breathing frequency (*D*_*F*_ ∼ *T*^2^). Thus, the increase in alveolar deposition becomes insignificant after certain duration of the breathing cycle. However, the alveolar deposition linearly increases with the breath hold time (*D*_*F*_ ∼ *T*^1.2^), which indicates that longer breath hold time can increase the chances of virus infection significantly.

Figure 11 shows that the effect of the breath hold time is more significant than that of the breathing frequency. Since both the curves diverge with time (refer to Fig. 11), it can be inferred that higher breath hold time can be dangerous in terms of getting infected from the virus laden droplets. The addition of lower breathing frequency (i.e., increase in inspiration and expiration time) can also cause the situation to worsen as it increases the residence time of the infected particles in the distal lung airways. The increase in the residence time will enhance the deposition in the alveolar airways since the diffusive deposition, which is the dominant mechanism in the distal lung, is directly proportional to time. Therefore, in addition to a lower breathing frequency, introduction of breath hold time in between inhalation and exhalation can increase the threat of virus infection in a crowded place.

## VI. SUMMARY

An experimental study of aerosol deposition has been carried out for a wide range of Reynolds numbers (10^−2^ ⩽ *Re* ⩽ 10^3^); for different capillary diameters, ranging from 0.3 mm to 2 mm, representing distal lung bronchioles; and for differing capillary aspect ratios. The aerosols were generated using an ultrasonic nebulizer with a mean droplet size of 6.5 *µ*m. The aerosol particles were doped with boron quantum dots, the deposition of which was quantified using a spectrofluorometer. The results conclude that dimensionless deposition in a particular bronchiole (*δ*) is inversely proportional to the aspect ratio of the bronchiole ($\bar{L}$) (refer to Fig. 6), but the effect of *Re* diminishes with increasing $\bar{L}$. The value of *δ* is found to increase exponentially with an increase in the dimensionless diameter ($\bar{D}$) for different $\bar{L}$. In addition, *δ* decreases with an increase in $\bar{L}$ for all $\bar{D}$. However, the variation of *δ* with $\bar{L}$ is small compared to its variation with $\bar{D}$. The value of *δ* is independent of the particle size based Reynolds number ($Re\bar{D}$). For all $Re\bar{D}$, *δ* exhibits an exponential increase with $\bar{D}$. $\delta \bar{L}$ is independent of $\bar{L}$ over several orders of magnitude of *Re*, which confirms the inverse relation between *δ* and $\bar{L}$ in Fig. 6. For low *Re*, $\delta \bar{L}∼Re^{-2}$, indicating that the amount of aerosol deposited is independent of the flow conditions and only depends on the aerosol conditions. This is the case with *diffusion* dominated deposition. The parameter regime where 10^−1^ < *Re* < 10 is identified as the zone where *sedimentation* is dominant. $\delta \bar{L}$ is independent of *Re* for 10 ⩽ *Re* ⩽ 10^3^, which is identified as the *impaction* regime. Figure 10 indicates that the lower breathing frequency or higher breath hold time in between inhalation and exhalation can increase the threat of virus infection in a crowded place since both of the phenomena increase the deposition in the alveolar region.

## Data Availability

The data that support the findings of this study are available from the corresponding author upon reasonable request.
